# The primary health care in the emirate of Abu Dhabi: are they aligned with the chronic care model elements?

**DOI:** 10.1186/s12913-017-2691-4

**Published:** 2017-11-14

**Authors:** Marília Silva Paulo, Tom Loney, Luís Velez Lapão

**Affiliations:** 1 Global Health and Tropical Medicine, Instituto de Higiene e Medicina Tropical, Universidade Nova de Lisboa, Lisbon, Portugal; 2 World Health Organisation Collaborating Center for Health Workforce Policy and Planning, Lisbon, Portugal; 3 Institute of Public Health, College of Medicine and Health Sciences, United Arab Emirates University, Al Ain, United Arab Emirates; 4 World Health Organisation Collaborating Center for Occupational Health, Al Ain, United Arab Emirates

**Keywords:** Abu Dhabi emirate, Health care surveys, Ambulatory healthcare, Health services research, Health personnel, Primary health care, United Arab Emirates

## Abstract

**Background:**

Abu Dhabi is the capital of the United Arab Emirates (UAE) and the largest of the seven emirates in terms of land mass and population. Abu Dhabi emirate has three different geographical regions: the Central Capital District, the Eastern Region, and the Western Region. The health system has been regulated by the Health Authority – Abu Dhabi (HAAD), and has been provided by the Abu Dhabi Health Services Company (SEHA), since 2007. The UAE has a high population-burden of morbidity and mortality related to chronic diseases. This paper aims to characterize the Primary Health Care (PHC) public services in Abu Dhabi using the Chronic Care Model (CCM) as a framework.

**Methods:**

Officially published data from HAAD, SEHA and the UAE Ministry of Health and Prevention was reviewed and abstracted. The Preferred Reporting Items Systematic Reviews and Meta-Analysis (PRISMA) statement was used as a baseline to review the PHC services through the CCM approach and to identify potential opportunities for improvement.

**Results:**

There are 38 SEHA Ambulatory Healthcare Centers (AHS) that provide PHC, from which 20 are located in the Eastern Region and the other 18 in the Central Capital District. The AHS adopted the principles of the patient-centered medical home model, aiming at providing structured, proactive and coordinated care. Implementation of the CCM elements aligns with those standards and is positively associated with the use of interventions targeting high-risk behaviors.

**Conclusion:**

The UAE has a strong foundation in place for addressing the growing problem of chronic diseases. The CCM has been shown to have beneficial effects on clinical outcomes reinforcing the PHC procedures and processes of care and should continue to inform systematic efforts to improve the care that lead to better lives for the Abu Dhabi community.

## Background

According to the World Health Organization, primary health care (PHC) are a core basis of the healthcare system. In 2008, the world report was launched named “Now more than ever” as a strategy to improve the access to primary care. Five years later, 2013, the world report focused in the “Universal health coverage”. There is still the need of discussion about the reorganization and follow-up of the health systems as there are many types of healthcare delivery configurations and discrepancies around the world [1, 2].

The United Arab Emirates (UAE) is a high-income developing country, the result of the aggregation of seven emirates: Abu Dhabi, Ajman, Dubai, Fujairah, Ras Al-Khaimah, Sharjah and Umm Al-Quwain.

**Fig. 1:**
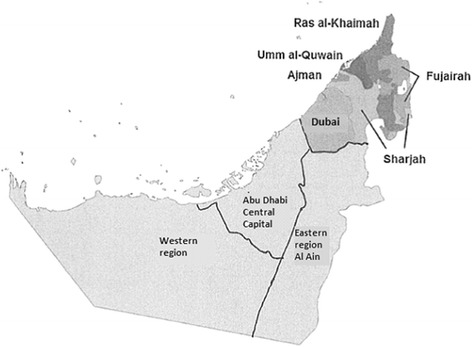
Geographical regions of the Abu Dhabi emirate (Adapted from Koornneef et al. BMC Health Serv Res. 2017;17(1):672. doi: 10.1186/s12913-017-2597-1.)

This study focuses only on the publically funded health system of the Emirate of Abu Dhabi. Abu Dhabi is the biggest emirate in population and land size performing almost 87% of the total area of the country [3] and it is also the capital of the country and where the government is based. Abu Dhabi has three different regions called the Abu Dhabi Central Capital region, the Eastern Al Ain region and the Western Al Gharbia region (Fig. 1).

**Fig. 2:**
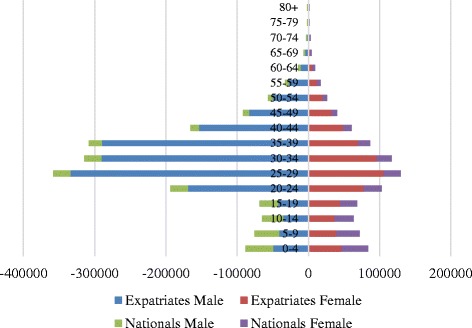
Population pyramid in the emirate of Abu Dhabi, in Mid 2014 (data from SCAD Yearbook 2015). Title: Population estimates by age group and gender in the Abu Dhabi emirate

The 2014 mid-year population estimate for the emirate of Abu Dhabi was 2,656,448 [4]. In this emirate, the majority of the population were male, with only a third (33.5%) comprised of women (see Fig. 2). The age group between 0 and 14 years constituted 16.9% of all the population, with the largest proportion aged between 15 and 64 years (82.1%) and only 1.0% of the population were aged ≥ 65 years [4] (see Fig. 2). Since 2000, there has been a slight decrease in the age group 0–14 years with a continuous increase in the age group 15–64 years, but in 2009–10 the trend was reversed.

The Abu Dhabi age population’s pyramid has an unusual shape and structure, showing a young population and a disproportion of males and females (see Fig. 2). This disproportion between genders in the Emirate of Abu Dhabi follows the country trend, a similar pattern to other developing countries in the Gulf Cooperation Council such as Qatar and Bahrain [5, 6]. The population structure of these countries is characterized by a predominantly young population and an expected equal ratio of males to females among the nationals. However, the unusual structure of the expatriate population is due to the mass recruitment of males employed in the industrial and construction sector and a heavy reliance on females in the service sector as domestic staff and shop workers. From the total of the population in Abu Dhabi, 66.5% were males, in 2014 [4].

**Fig. 3:**
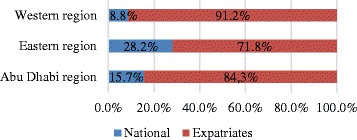
Population by Abu Dhabi emirate region and nationality. Title: Population by region and nationality

There is an unequal distribution among UAE nationals and expatriates, not just regarding the age of the population, as well as regarding the regions. Only 19.0% of the total population living in Abu Dhabi are UAE nationals. Over half (52.0%) of UAE nationals live in the Abu Dhabi Capital Center, 42.0% live in the Eastern region of Al Ain and 6.0% live in the Western region (Fig. 3). Expatriates comprise 81.0% of the population in the emirate of Abu Dhabi and 64.0% of them live in Abu Dhabi Capital Center region [4].

These demographic characteristics influence the distribution and the delivery of the healthcare services that should be community-oriented to provide a continuous and coordinated care meeting the health needs of the population. The population of nationals is young, but they will be old and chronic diseases are the leaders among them. The healthcare system must be prepared for it. Although the health insurance is mandatory for all the residents in the country, it is difficult to maintain a continuous care in the community for the expatriates. This is due to the nature of the expatriates and the high turn-over. The PHC is care in the community *per definition* so the programs tend to be more centered in the permanent population – the UAE nationals. This also affects the distributions of the PHC between the regions of the emirate.

The main causes of mortality in Abu Dhabi, in 2013, were diseases of the circulatory system (37.0%), other causes (24.0%), external causes of morbidity (16.0%), cancer (16.0%), endocrine, nutritional and metabolic diseases (2.0%), congenital malformations, deformations and chromosomal abnormalities (2.0%) and injury, poisoning and certain other consequences of external causes (3.0%) [7]. In UAE, in the same year, it was estimated that non-communicable diseases account for 65.0% of all deaths (9700 people died) and the probability of dying between 30 and 70 years from the non-communicable diseases was 19.0% [8].

### Health system

In 2007, the Abu Dhabi government created two organizations: the Health Authority – Abu Dhabi (HAAD) (Abu Dhabi Law No. 1 of 2007) and the Abu Dhabi Health Services Company (SEHA) (Abu Dhabi Amiri Decree No. 10 of 2007) [7].

The HAAD is responsible for the definition of health system strategies, evaluation, and analysis of health issues of the population and performance of the system regulating all healthcare actors (public/private, provider/payer/professionals). The aims of HAAD are to achieve, develop, follow-up and monitor high standards in health, curative, preventive, medical services and health insurance in the Emirate of Abu Dhabi and to keep high international standards [7]. The HAAD has a healthcare strategy with clear priorities and goals and its own public health programs.

SEHA (the Arabic word for health) is responsible for operating the publicly funded health system of Abu Dhabi and to upgrade and deliver world-class healthcare. The purpose of SEHA is to provide comprehensive healthcare services in cities and in rural areas, with the latest technology and treatments and technical and medical specialists in all facilities. SEHA owns and manages the public healthcare system of the emirate and upgrades and improves healthcare delivery based on international standards and external accreditation [8].

The aim of this paper is to systematically review the available information and data to characterize and analyze the PHC public services in the emirate of Abu Dhabi in the United Arab Emirates using the Chronic Care Model as a framework.

### Chronic care model

The propose of the Chronic Care Model (CCM) model is to have a population-based daily care for all with a structured and planned team care interventions [9]. It is a multicomponent model that integrates six elements which facilitate high-quality care and each element of the model has its own strategies (Table 1) and development concepts that can be applied to a variety of healthcare settings, chronic diseases and target populations [9].

**Table 1:** Chronic Care Model elements and respective strategies

Model Elements	Approaches/Strategies
Self-Management Support	- Emphasize the patient’s central role in managing their health - Use effective self-management support strategies that include assessment, goal-setting, action planning, problem-solving and follow-up - Organize internal and community resources to provide ongoing self-management support to patients
Community	- Encourage patients to participate in effective community programs. - Form partnerships with community organizations to support and develop interventions that fill gaps in needed services. - Advocate for policies to improve patient care
Health System	- Visibly support improvement at all levels of the organization, beginning with the senior leader. - Promote effective improvement strategies aimed at comprehensive system change. - Encourage open and systematic handling of errors and quality problems to improve care. - Provide incentives based on quality of care. - Develop agreements that facilitate care coordination within and across organizations
Delivery System Design	- Define roles and distribute tasks among team members. - Use planned interactions to support evidence-based care. - Provide clinical case management services for complex patients. - Ensure regular follow-up by the care team. - Give care that patients understand and that fits with their cultural background
Decision Support	- Embed evidence-based guidelines into daily clinical practice. - Share evidence-based guidelines and information with patients to encourage their participation. - Use proven provider education methods. - Integrate specialist expertise and primary care
Clinical Information System	- Provide timely reminders for providers and patients. - Identify relevant subpopulations for proactive care. - Facilitate individual patient care planning. - Share information with patients and providers to coordinate care. - Monitor performance of practice team and care system.

## Methods

This was a systematic review designed to contribute to the knowledge of the primary healthcare services in Abu Dhabi, and investigate whether the aims of the PHC in Abu Dhabi were aligned with the six elements from the Chronic Care Model. To perform this, a systematic review was conducted to synthesize the facilities available to deliver primary healthcare to the populations and which guidelines and models are followed and implemented to address the chronic diseases problem.

A systematic literature review attempts to “collect all empirical evidence that fits pre-specified eligibility criteria in order to answer a specific research question”. Systematic reviews have five key characteristics according to the Cochrane Handbook for Systematic Reviews: (i) a clear set of objectives with pre-defined eligibility criteria; (ii) an explicit and reproducible methodology; (iii) a systematic search to identify all studies that would meet the eligibility criteria; (iv) an assessment of the validity of the findings; and (v) a systematic presentation of the characteristics and findings of the studies [10].This study follows the Cochrane Collaboration guidelines for conducting systematic reviews and the focus was the specific research question: “What are the PHC in Abu Dhabi Emirate?” and “Are the aims of the PHC properly aligned with CCM approaches? If they are not, where are the gaps?”

### Data sources

#### Eligibility criteria

This review was performed based on secondary data sources that are available in the public domain. The search was limited to English language journals or reports. The specific search terms and strategy are displayed in Table 2. Only recently published reports and data that was no more than three years old (i.e. before 2013) were included in the review.

**Table 2:** Study selection information sources

Information sources		Search strategy	Studies found
Data bases	PubMed and manual search: Google Scholar	“United Arab Emirates” AND “Abu Dhabi” AND “Primary Health Care” AND “Chronic Care Model”	“A successful chronic care program in Al Ain-United Arab Emirates”
			“Effectiveness of chronic care models: opportunities for improving healthcare practice and health outcomes: a systematic review”
		“United Arab Emirates” AND “Primary Health Care” AND “Chronic Care Model”	“The “Arab World” is Not a Useful Concept When Addressing Challenges to Public Health, Public Health Education, and Research in the Middle East”
			“Population structure and the burden of disease in the United Arab Emirates”
			“Healthcare Regulation in the United Arab Emirates. The Health Authority – Abu Dhabi”
		“United Arab Emirates” AND “Abu Dhabi” AND “Health System”	“Health and health system performance in United Arab Emirates”
			“Financing health care in the United Arab Emirates.”
			“Health systems in United Arab Emirates: progression, challenges and future directions”
		“Chronic Care Model” AND “aims”	“Improving Chronic Care: *The “Guided Care” Model*”
			“Evidence on the chronic care model in the new millenium”
			“Does the Collaborative Model Improve Care for Chronic Heart Failure?”
			“Improve Chronic Illness Care: Translating Evidence Into Action”
			“Interventions to Improve the Management of Diabetes in Primary Care, Outpatient andCommunity Settings: A Systematic Review”
			“Rethinking Prevention in Primary Care: Apllying the Chronic Care Model to Address Health Risk Behaviors”
		“Patient-Centered Medical Homes”	“Evidence Showing Effectiveness of NCQA Recognition: Benefits of the Patient-Centered Medical Home”
			“Patient-centered medical home demonstration: a prospective, quasi-experimental, before and after evaluation.”
			“Value and the Medical Home: Effects of Transformed Primary Care”
			“Is patient-centered care the same as person-focused care?”
Metaresources of infroamtion	MOHP		Healthcare facilities
	HAAD	Revision of Annual Health Reports	Health Statistics 2012
			Health Statistics 2013
		Direct search about relevant topics	Book 1: legislation Establishing the Health Sector
			Public Health Priorities and Goals
			Public Health Programs
	SEHA	Revision of Annual Health Reports	Annual Report 2013
		Direct search about relevant topics	Wy SEHA exists
			Mission, Vision and Values
			JCI Acredition
			Ambulatory Healthcare Systems
	SCAD	Revision of Annual Health Reports	Statistical yearbook 2015
	WHO	Revision of Annual Health Reports	World Health Statistics - 2013
			World Health Statistics - 2015
		Direct search about relevant topics	United Arab Emirates - country profile
			Non-Comunicable Profile - UAE
			Country Cooperation Strategy at glance - UAE

#### Information sources

The data collection was obtained through the consultation of bibliographic databases and meta-resources of information as the World Health Organization, World Bank, Institute of Health Metrics and Evaluation, UAE Ministry of Health and Prevention, HAAD, SEHA, and bibliographic references of the official reports obtained. This stage went from September 2014 to March 2016.

#### Search/study selection

The primary search on Google Scholar and PubMed included the following medical subject headings: “United Arab Emirates”, “Abu Dhabi”, “Health System”, “Primary Health Care” and “Chronic Care Model”. Some references of the articles that we found interesting were also screened and a manual search was conducted in all the health related UAE official websites: Ministry of Health and Prevention, HAAD, SEHA, Dubai Health Authority, Statistics Center of Abu Dhabi.

#### Data collection process/data items

Relevant data were independently extracted from available reports and reviewed considering the study PICOs (population, intervention, comparisons, and outcomes) [11]. The population under study and the comparisons should be from UAE or same geographic regions and the interventions and outcomes relevant to primary healthcare or chronic diseases settings.

## Results

### Studies selection

**Fig. 4:**
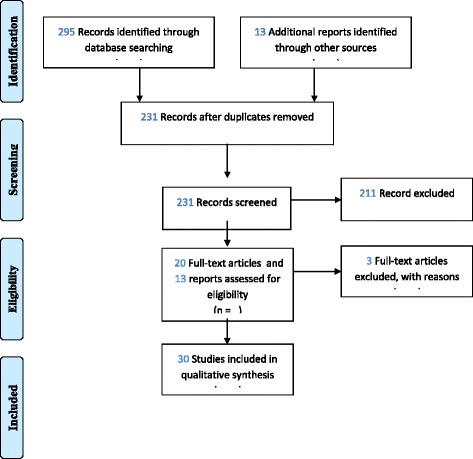
PRISMA Flow Diagram

With the utilization of the selection strategy described above, it was found 295 studies in the databases and another 13 reports were considered important. 231 titles and abstracts were screened and 211 were excluded, mainly due to the population (we found lots of studies from another Arab country and specifically addressing one health conditions). The studies and 13 reports were selected to analyze and it was felt the need of a search for some extra articles included in the references of the articles we analyzed, so at the end, we reached the number of 20 articles and 13 reports (Fig. 4). After screening their eligibility criteria, we have included 33 studies and reports in our qualitative analysis. Three of them were not used due to the studies specifications, like an economic vision of the healthcare system. Table 2 presents the summary of the relevant literature included.

### Synthesis of results

#### Health workforce

The health workforce is one of the key components of the health system and between 2007 and 2013 the UAE’s health system had an active health workforce density of 25.3 physicians, 31.6 nursing and midwifery personnel, 4.3 dentists and 5.9 pharmacists per 10,000 population [12]. During the period 2005–2012, the physician density increased from 19.3 per 10,000 population, while the nursery and midwifery personnel decreased from 40.9 per 10,000 population, and the dentists and pharmacists remained approximately the same [13]. In 2013, the Emirate of Abu Dhabi presented high ratios of physicians (25.1) and of nurses (52.1) per 10,000 population [14]. Translating these figures into absolute numbers, there were 6864 physicians, 1220 dentists, 14,235 nurses, 5332 allied health professionals and 2396 pharmacists divided by 1626 licensed healthcare facilities (public and private) [15] From the 6864 in the emirate, 68.83% are in Abu Dhabi Capital Center, 26.38% are in the Eastern region and 4.79% are in the Western region [15].

#### SEHA

Services is a SEHA Health System Facility which is responsible for manage the facilities in a community-based services across the emirate of Abu Dhabi [16]. SEHA is responsible and operates 12 hospitals, 11 dialysis centers, 62 ambulatory healthcare centers and clinics, and 2 blood banks [17], employing a total of more than 17,500 professional staff across all three geographical regions of the Emirate of Abu Dhabi (Fig. 1) [16].

#### Hospitals

In 2002, there were 35 public hospitals in the UAE, as well as 14 private hospitals and 128 outpatient clinics. At this time, although several small private hospitals have been set up over the past years [3] there was still a tendency for UAE nationals to seek treatment aboard in the United States and Europe. In Abu Dhabi, there were 41 private and public hospitals (10% of all are SEHA’s), 587 healthcare centers (13% of all are SEHA’s), 335 clinics (1% of all are SEHA’s) and 402 pharmacies (14% of all are SEHA’s) in 2013 [15]. During the same year, the private PHC centers and clinics attended more outpatients (5,034,581) comparing with the public ones by SEHA (2,339,645) [17].

#### Ambulatory healthcare services (AHS)

The Joint Commission International (JCI) is considered the gold standard in global health care and is part of a global enterprise of dynamic and non-profit organizations that lead the innovative solutions to help health care organizations improving performances and outcomes. The focus of JCI is to “identify, measure and share best practices in quality and patient safety with the world.” It In 2012, the AHS was recognized by the JCI with ‘Gold Seals’, becoming the first institution of this type with such accreditation, in the world [18]. All of the ambulatory healthcare centers and clinics of the eastern region were part of this accreditation scheme [19]. To achieve this reward, these facilities must fulfill specific standards, specific for its own type, in this case – Ambulatory Care, and must address international patient safety/infection control goals, patient access, and assessments, patient records, and information flows and certain administration procedures [20]. These standards are validated by JCI.

From the 62 ambulatory healthcare centers, only one is an occupational healthcare center, 10 are disease prevention and screening centers (primarily for infectious disease residency visa screening), and 9 dialysis centers and clinics [16]. The AHS is also responsible for the Mobile Health Clinics and the School Health Services, which encompasses 298 school clinics (including higher schools, universities and private schools) [17]. Approximately 80% of all AHS patients visits are PHC consultations [17] with diabetes and cardiovascular disease comprising the majority of the chronic disease case load managed in these centers [21].

#### Primary health care

The AHS, under SEHA, has 38 clinics. From those, 18 facilities are in the region of Abu Dhabi and 20 facilities are in the Eastern region (Al Ain). The AHS does not provide PHC services in the Western region [22].

#### Primary health care/AHS

**Fig. 5:**
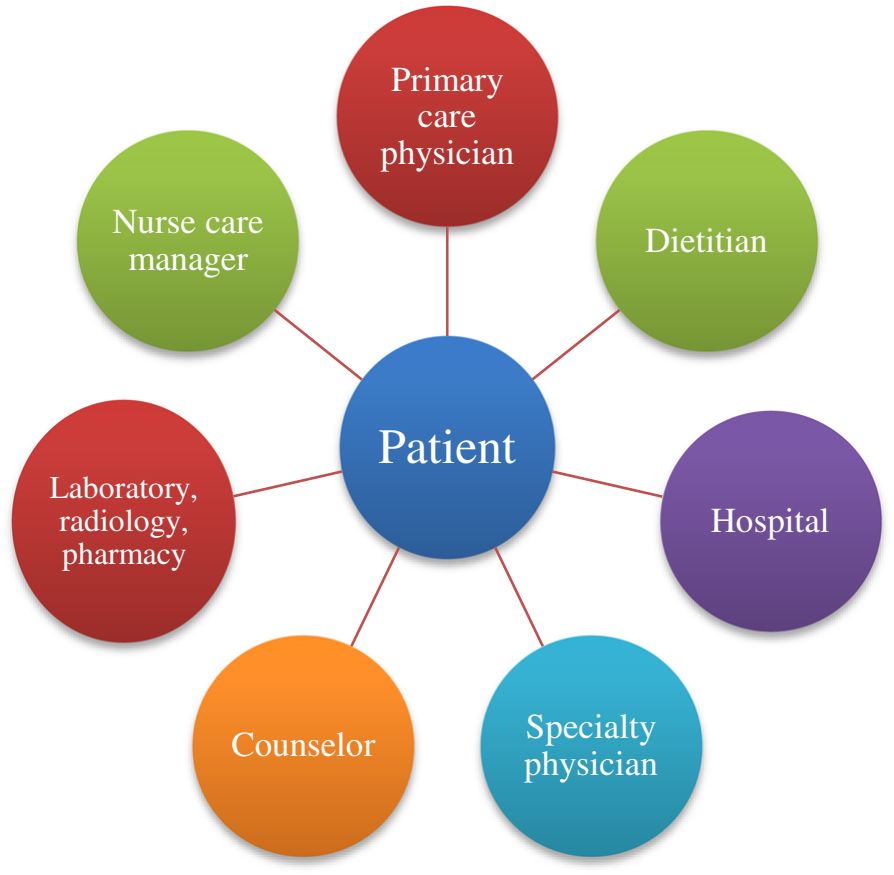
Patient-centered medical home model [17]

In 2013, the PHC under AHS adopted the Patient-Centered Medical Home model and principles. This model has the same core foundation as the CCM, with the main objective to provide structure, proactive, and coordinated care for patients rather than episodic treatments for illnesses [17]. As the name of the model suggests, it has the patient in the center surrounded by the primary care physician, the dietician, the nurse case manager, the counselor, the specialty physician, the laboratory, radiology, pharmacy, and the hospital (Fig. 5).

Globally, the this model is influencing some of the reforms in PHC delivery, it was vital to the primary care reform in USA [23]. There is evidence that this model is improving patient outcomes, moderating health disparities and reducing health resources by reducing the number of hospitals visits. The Patient-centered medical home aims to inspire quality care, engage patients and expand access and delivery options [24]. These aims are aligned with the CCM ones and there is more than one study evaluating the Patient-centered medical home interventions, such as patient registers, care plans, e-mails between the patient and health professional (team), self-management strategies and population profiles [23, 25, 26]. These Patient-centered medical home interventions can be easily linked to the CCM elements: clinical information systems, delivery system design, self-managements, and community. The Patient-centered medical home model was only recently implemented in the Abu Dhabi emirate in 2013 and there are currently no publicly available records or reports on the monitoring or evaluation process.

### CCM aims

The CCM was designed with the main goal of transforming the daily care routine of the patients with chronic conditions from acute and reactive to proactive, planned and population-based [27]. SEHA developed a Health Education program to help to transform the routine of the patients with Diabetes, Heart and Circulation Diseases, Kidney Diseases and Health Lifestyle. Conferring with the 2012 Heath Statistics of Abu Dhabi “There are no systems in place to support patient self-care and management of chronic disease” [14], however, we found some evidence that it exists. Table 3 shows the evidence that we collect for each element of the CCM.

**Table 3:** CCM elements evidence in AHS

Model Elements	Evidence in Primary Healthcare Centers	Gaps
Self-Management Support	Through Health Education in SEHA’s website and App: - Information about the importance of patients decisions and daily routines that affect their health and specifically according with the disease; - Information available about how to manage the types of Diabetes, Heart and Circulation Diseases, Kidney Diseases and Health Lifestyle	Use effective self-management support strategies that include assessment, goal-setting, action planning, problem-solving and follow-up
Community	- SEHA is establishing electronic programs and communication channels (interactive when possible). - In 2013, lectures on health and nutrition were delivered for family foundation development schools.	- Encourage patients to participate in effective community programs; - Form partnerships with community organizations to support and develop interventions that fill gaps in needed services; - Advocate for policies to improve patient care
Health System	- In 2013 SEHA launched the National Hospital Quality Measure; - Provides performance data to the different professions; - Set out procedures that will create shared responsibility for individuals towards their duties. - Provides incentives based on quality of care; - Names and rewards outstanding individual contributors in different categories; - Each SEHA’s hospital have linked Ambulatory Healthcare Services; - SEHA establishes partnerships with healthcare providers to ensure the accessibility (e.g. Jonh Hopkins Hospital, Cleveland Clinic)	- Promote effective improvement strategies aimed at comprehensive system change; - Visibly support improvement at all levels of the organization, beginning with the senior leader;
Delivery System Design	- Care Plans; - Give care that patients understand and that fits with their cultural background	- Define roles and distribute tasks among team members; - Use planned interactions to support evidence-based care; - Provide clinical case management services for complex patients; - Ensure regular follow-up by the care team;
Decision Support	- In 2013, SEHA launched “Kafu”, consumer care development program to standardize costumer care by adopting the best practice; - Provides useful and specialized data; - SEHA offers interactive tutorials, videos, PDF’s and quizzes about the topics in Health Education; - Integrative teams with specialist expertise in primary care (ex: Dieticians following up diabetes patients in ambulatory centers)	- Embed evidence-based guidelines into daily clinical practice; - Share evidence-based guidelines and information with patients to encourage their participation;
Clinical Information System	- In case of patients with health disease SEHA facilitates an emergency plan that the patient must know; - The patients information is available in SEHA database and any clinic can see it when its needed; - The PCMH dash board has graphs, charts and spreadsheets about chronic disease patients and doctors performance;	- Identify relevant subpopulations for proactive care; - Provide timely reminders for providers and patients; - Share information with patients and providers to coordinate care; - Monitor performance of practice team and care system.

## Discussion

### Summary of evidence

The UAE has experienced a profound change from an underdeveloped region of small desert principalities to a modern state. The major transformation started in 1973–74 with the extraction and exportation of oil, and wise investments of the UAE leadership. Nowadays the UAE is a high-income developing country with a competitive health system, which has been earning multiple international awards. The healthcare is regulated at both Federal and Emirate level (various entities at Emirate level) which mean that the division of power and regulatory entities are sometimes unclear in certain areas, as in relation to licensing and monitoring/control medical institutions [28]; however, in Abu Dhabi emirate there is just one institution, HAAD, responsible for it. According to the 2013 Health Statistics of Abu Dhabi, there was an unequal distribution in specialty care across the three geographical regions of the Emirate of Abu Dhabi. Rural primary care is not well developed in the emirate, especially in the Western region and need several improvements like solving the critical shortages.

The UAE healthcare system is faced with a rising demand due to high population caused by natural growth and positive net migration. Moreover, the UAE national population is young but with high rates of chronic disease risk factors; therefore, the rates of chronic disease are projected to increase greatly as the young population ages.

The Patient-centered medical home adopted by SEHA to the AHS has the same base of care as the CCM. The MacColl Center for Health Care Innovation developed the Primary Care Team Guide to help the leaders and engage the staff transforming to a more effective team. “Team-based care can enhance the support and health outcomes of individuals with chronic diseases and be integral to implement the CCM and becoming a Patient-centered medical home” [29].

One of the challenges of the current model of care is the lack of sufficiently self-management support and prevention (screening programs and diagnostic services are not integrated into care plans) and patients have undirected access to services and specialty care increasing inappropriate use and over-supply of services [14]. Although this review found evidence of patient’s central role in managing their health, as well as community resources available to provide on-going self-management to support the patients.

The focus of Abu Dhabi’s model of care is on empowering the patients, which is a key focus of the components of the CCM as well. There is evidence that the strategies in place in the primary healthcare of this emirate are linked and share what the CCM defines as better approaches to each of its elements. The future challenges will address the need to put in practice the conceptualization between the chronic disease/condition center to the patient and focusing on the team dynamics.

### Limitations

The limitations of this study are the lack of recently available data and monitoring/evaluation of data publicly available. Also, the continuous need for research in the fast developing countries with such a different pattern of nationalities and regulation entities.

## Conclusions

The Abu Dhabi emirate health system is internationally well-positioned and competing with others from the developed countries, even facing the challenge of the unique characteristic of the population, both UAE citizens, and expatriates. It seems there is an effort in following the latest scientific evidence with the intention to achieve health gains (patient outcomes and limited resources) economic reductions. The HAAD aims at promoting a future healthcare system based on empowered patients. To achieve this goal, pro-active check-ups and convenient routine follow-up should help to prevent disease and must be implemented in well-developed primary and sub-acute care (with home care and integrated telemedicine).

The adoption of the Patient-centered medical home was undoubtedly a strategic choice and the model seems to be aligned with the CCM. Now that three years have passed since the implementation of the Patient-centered medical home, there is the need to monitor and oversee its implementation and evaluate the short-term results. It will be fundamental to conduct further research to understand some questions like “how health professionals are adapting to changes in care? Do the patients already notice significant differences in the service provided?” that can lead us to achieve even more improvements in the health system of Abu Dhabi emirate.

## Abbreviations

AHS: Ambulatory Healthcare Services
CCM: Chronic Care Model
HAAD: Health Authority Abu Dhabi
PHC: Primary Health Care
SEHA: Abu Dhabi Health Services Company
UAE: United Arab Emirates
